# Hot receptors in the brain

**DOI:** 10.1186/1744-8069-2-34

**Published:** 2006-11-08

**Authors:** Hendrik W Steenland, Shanelle W Ko, Long-Jun Wu, Min Zhuo

**Affiliations:** 1 Department of Physiology, Faculty of Medicine, University of Toronto, Toronto, Canada

## Abstract

Two major approaches have been employed for the development of novel drugs to treat chronic pain. The most traditional approach identifies molecules involved in pain as potential therapeutic targets and has focused mainly on the periphery and spinal cord. A more recent approach identifies molecules that are involved in long-term plasticity. Drugs developed through the latter approach are predicted to treat chronic, but not physiological or acute, pain. The TRPV1 (transient receptor potential vanilloid-1) receptor is involved in nociceptive processing, and is a candidate therapeutic target for pain. While most research on TRPV1 receptors has been conducted at the level of the spinal cord and peripheral structures, considerably less research has focused on supraspinal structures. This short paper summarizes progress made on TRPV1 receptors, and reviews research on the expression and function of TRPV1 receptors in supraspinal structures. We suggest that the TRPV1 receptor may be involved in pain processing in higher brain structures, such as the anterior cingulate cortex. In addition, some regions of the brain utilize the TRPV1 receptor for functions apparently unrelated to pain.

## Background

The TRPV1 (transient receptor potential vanilloid-1) receptor was originally isolated using a calcium imaging-based expression method [1-3]. This receptor is activated by capsaicin (the pungent ingredient of hot peppers), protons, and heat (>43°C), and behaves as a non-selective cationic channel with high permeability for calcium [2-6]. A number of endogenous ligands suggested for these receptors include: protons, ATP, lipoxygenase products, anandamide, N-oleoyldopamine, and N-arachidonoyl dopamine [5-7].

TRPV1 receptor subunits are predicted to have six transmembrane spanning domains with an intramembrane loop connecting the 5th and 6th domains [3]. A variety of molecules and proteins interact with and/or modulate the TRPV1 receptor. These include: TRPV3 and phosphitidylinositol-4,5-bisphosphate receptor modulation of capsaicin binding [8,9] and interactions with scaffolding and synaptic vesicle proteins [10,11].

The role of the TRPV1 receptor in pain-related behaviors has been demonstrated with gene knockout mice [12]. Specifically, these mice showed impairments in their ability to detect painful heat stimuli, and demonstrated little thermal hypersensitivity during an inflammation test [12]. Responses to noxious mechanical stimulation were not altered by the gene knockout, suggesting a selective modality for the TRPV1 receptor [12]. While this study demonstrates that TRPV1 of the dorsal root ganglia (DRG) neurons modulate nociceptive behaviors, the role of the TRPV1 in supraspinal brain structures was not investigated.

Most research on the TRPV1 receptor has been conducted on spinal cord and peripheral structures [6,13]. In this review, we highlight studies that report the expression and function of the TRPV1 receptor in supraspinal structures, with particular emphasis on brain regions involved in the processing of pain.

## Expression in the brain

A variety of studies have been conducted to determine the expression profile of the TRPV1 receptor throughout the brain [3,14-21] (See additional file 1: Table 1). Initial studies with [^3^H] resiniferatoxin (RTX), which label TRPV1 receptors, have shown that TRPV1 is expressed in the trigeminal ganglia and DRG [15]. However, no TRPV1 receptor expression was detected in the brain. Confirmation of these findings was obtained with northern blot analysis [3]. Collectively, these results suggest that the TRPV1 receptor is not expressed in the brain.

**Figure 1:**
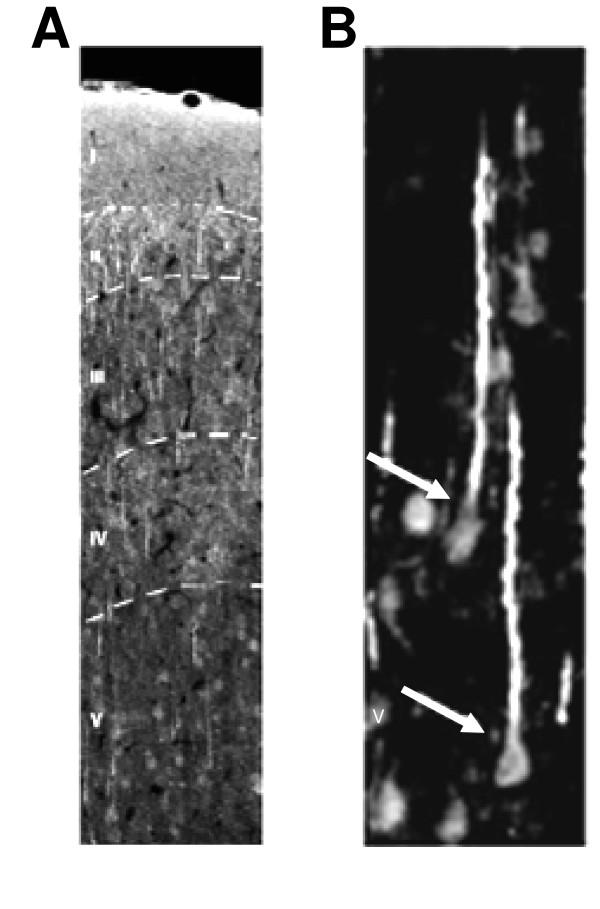
**Expression of TRPV1 in the cortex**. A. Cortical immunostaining for the TRPV1 receptors in the rat cortex, with each layer indicated (I-V). B. Enlargement of cortical layer V (from A) with arrows indicating pyramidal cells. Reprinted from [19] ^© ^2005 with permission from Elsevier Science.

Acs et al. [17] established that TRPV1 receptors could be detected in the preoptic hypothalamus, locus coeruleus, and ventral thalamus of human and rat brain, using a modified RTX-labeling technique. The existence of TRPV1 receptors in the brain has been supported numerous times with assays for both TRPV1 protein and mRNA [14,16,18-22] (for example, Fig. 1). One of the most comprehensive studies was conducted by Roberts et al. [18] in which RTX labeling in the brain of TRPV1 knockout mice was compared to mice with the TRPV1 gene. This study revealed a wide distribution of TRPV1 receptors in the brain, including regions of the cerebral cortex, cerebellum and a variety of subcortical structures. It should be emphasized that TRPV1 RNA is approximately 28 times greater in the DRG than in any other brain region [21]. Thus, the most likely explanation for the absence of TRPV1 detection in the brain, described by other investigators [3,15], is that their assays were not sensitive enough to detect the lower expression in the brain.

The TRPV1 receptor is localized to neuron cell bodies and dendrites, astrocytes, and perivascular structures within the brain [14,16,18,19]. TRPV1 can be detected predominantly on postsynaptic spines at the subcellular level [19]. In addition, TRPV1 can be detected in pericytes and at the feet of astrocytes surrounding small vessels [19]. It has also been demonstrated that TRPV1 co-localizes and physically interacts with TRPV2 receptors within the brain [14]. These two receptors were found to be extensively co-localized within the cytoplasmic component and plasma membrane of cortical neurons [14]. In contrast, there was much less co-localization of TRPV1 and TRPV2 receptors reported for DRG neurons [14]. Another point of departure between TRPV1 expression in the DRG and the brain is that, neonatal capsaicin treatment in rats reduces expression of TRPV1 mRNA in the DRG, but not in the brain [16]. The authors suggested that neonatal capsaicin treatment may deplete neurotrophic factors which are required for the survival of peripheral neurons and not central neurons [16]. A more recent finding demonstrated that a high dose of capsaicin to cultured mesencephalic dopaminergic neurons induces cell death [23]. The cell death was likely a consequence of calcium entry leading to mitochondrial damage [23]. Thus capsaicin can destroy central neurons, suggesting that neonatal capsaicin treatments may not reach toxic levels in supraspinal structures.

Since the TRPV1 receptor is activated by capsaicin and is involved in nociceptive processing at the level of the DRG and sensory nerve endings, it is likely that this receptor occupies a role in pain processing at other brain regions. Such regions might include: the rostral ventromedial medulla, periaqueductal grey, solitary tract nucleus (NTS), preoptic hypothalamus, ventral thalamus, somatosensory cortex, anterior cingulate cortex (ACC), and insula [24,25]. Indeed, the TRPV1 receptor and its mRNA have been localized to neurons in most of these regions (See additional file 1: Table 1.). However, regions such as the NTS appear to express more TRPV1 receptors than that of cingulate or somatosensory cortices. Considering that the expression of DRG TRPV1 receptors can be up-regulated in response to peripheral inflammation [26], it would be of interest to determine whether TRPV1 receptors of the ACC and somatosensory cortex can be similarly regulated.

## TRPV1 receptor activation in the brain

The effect of TRPV1 receptor activation in a variety of brain regions has been investigated. These areas include the following: the ventral medulla, periaqueductal grey, solitary tract nucleus (NTS), dorsal raphé nucleus, locus coeruleus, hypothalamus, thalamus, ventral tegmental area, substantia nigra, hippocampus, cerebellum and cortex. Many of these studies have utilized pharmacological activation of TRPV1 receptors with capsaicin. In this section, the effect of TRPV1 receptor activation in the central nervous system will be examined and possible synaptic mechanisms will be addressed.

### Ventral medulla

The TRPV1 receptor is involved in the modulation of cardiovascular, respiratory, and temperature control systems at the level of the ventrolateral medulla [27,28]. Microinjection of capsaicin (0.5–50 nmol) into the ventrolateral medulla at the level of the hypoglossal nerve roots was found to increase respiratory output, arterial pressure, and heart rate in an anesthetized rat preparation [27]. Infusion of capsaicin into more caudal regions reduced arterial pressure and heart rate in both anesthetized and chronic rat preparations [27].

Acute systemic administration of capsaicin results in an initial hypothermia, followed by hyperthermia [28-30]. The hypothermia is a result of enhancement of autonomic heat loss mechanisms (e.g. peripheral vasodilation) and a depression of heat saving mechanisms (e.g. shivering) [31]. The latent hyperthermia is likely a result of sympathoadrenal activation [29,32]. Osaka et al. [28] found that lesions of the rostral ventrolateral medulla, a region containing sympathoadrenal preganglionic neurons, largely attenuated capsaicin-induced hyperthermia in anesthetized rats. Consistently, microinjection of capsaicin (500 μmol) into the rostral ventrolateral medulla was found to elicit hyperthermia [28]. The results suggest that capsaicin activates the rostral ventrolateral medulla which then increases sympathoadrenal activation leading to heat production. It will be of interest to determine whether the actions of capsaicin in this region are specific to the TRPV1 receptor.

### Periaqueductal grey

The periaqueductal grey is a well established component of the pain modulatory circuitry and projects to the rostral ventromedial medulla [25,33]. The rostral ventromedial medulla can subsequently exert descending modulation over nociceptive spinal reflex pathways [33-37]. Palazzo et al. [38] demonstrated that capsaicin injection (1–6 nmol) at the periaqueductal grey can increase the latency of nociceptive responses, indicating analgesia. This effect could be blocked by local antagonism of NMDA and metabotropic glutamate receptors. In contrast, McGaraughty et al. [39] found that capsaicin (10 nmol) injected into the dorsal periaqueductal grey could decrease the latency of both nociceptive behavioral responses and rostral ventromedial medulla tail-flick-on cell activity, suggesting hyperalgesia. To account for this discrepancy, McGaraughty et al. [39] suggested that the fast delivery of capsaicin by Palazzo et al. [38] may have desensitized the TRPV1 receptors resulting in analgesia.

A recent investigation by Maione et al. [40] demonstrated that elevation of endocannabinoid levels, with an inhibitor of fatty acid amide hydrolase, in the ventrolateral periaqueductal grey can produce analgesia and hyperalgesia. This effect was shown to be dependent on the activation of TRPV1 and CB1 (cannabinoid receptor-1) receptors. Low doses of inhibitor at the periaqueductal grey produced rapid hyperalgesia. The hyperalgesia was proposed to result from an increase in 2-arachidonoylglycerol, which stimulates CB1 receptors preferentially over TRPV1 receptors [40]. This leads to descending inhibition of off-cells and stimulates on-cells in the rostral ventromedial medulla, speeding up nociceptive responses [40]. Higher doses of fatty acid amide inhibitor cause rapid analgesia followed by a delayed hyperalgesia [40]. The findings were explained by suggesting that anandamide levels increase and stimulate TRPV1 receptors resulting in analgesia. Subsequently, 2-arachidonoylglycerol levels increase to stimulate CB1 receptors resulting in hyperalgesia. Again, the effects of the inhibitor at the periaqueductal grey would be mediated through descending modulation of the appropriate rostral ventromedial medulla circuitry [40]. Consistent with the concept that these two receptors modulate descending facilitatory and inhibitory output to the rostral ventromedial medulla, Maione et al. [40] found that some neurons of the periaqueductal grey co-expressed TRPV1 and CB1 receptors.

### Nucleus of the solitary tract

Activation of TRPV1 receptors in the NTS has been found to induce hypotension, bradycardia [41], and reduction of respiratory rate [42]. In-vitro brainstem slice experiments demonstrated that acute capsaicin (100 nM) treatment induces a rapidly developing inward current in NTS neurons. Capsaicin treatment also enhanced spontaneous glutamatergic currents [43]. The effects of the capsaicin treatment were restricted to a subpopulation of NTS neurons [43]. Additional evidence suggested that the capsaicin was acting on presynaptic TRPV1 receptors to enhance glutamate release onto AMPA receptors [43]. In addition, capsaicin sensitive neurons of the NTS, but not insensitive neurons, can be characterized by large transient outward currents [44]. The results suggest that within a particular brain region, activation of TRPV1 receptors may selectively affect neurons characterized by distinct electrophysiological properties.

### Dorsal raphé nucleus

Peripheral administration of capsaicin results in bursting activity in the dorsal raphé nucleus, recorded with intracortical electroencephalogram in rats [45]. In addition, direct injection of capsaicin (65 nmol) into the dorsal raphé nucleus increases vasodilation of the skin and decreases core body temperature in anesthetized rats [46].

### Locus coeruleus

The locus coeruleus is activated by painful stimuli and is involved in the production of antinociception [33,47]. Intravenous administration of capsaicin increases firing rates of locus coeruleus neurons in anesthetized rats [48]. This increased firing even occurred following neonatal capsaicin treatment to destroy sensory nerve fibers, indicating a central effect of capsaicin [48]. Consistent with this excitatory effect, TRPV1 activation at the locus coeruleus with capsaicin (1 μM) was found to enhance glutamatergic miniature excitatory postsynaptic currents through a presynaptic mechanism [49].

### Hypothalamus

**Figure 2:**
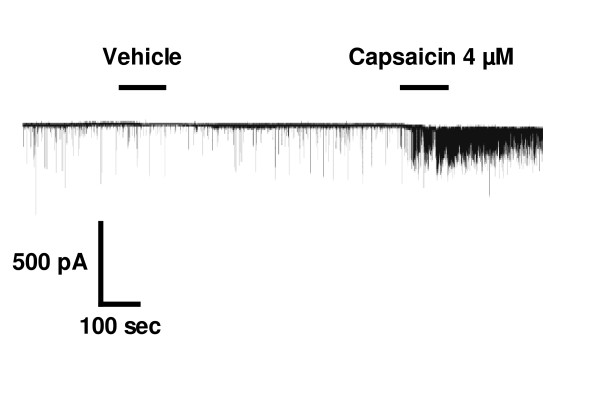
**TRPV1 modulates synaptic events in the hypothalamus**. Activation of TRPV1 receptors increases the frequency of postsynaptic currents of hypothalamic medial preoptic nucleus neurons studied in-vitro. Reprinted from [51] ^© ^2005 with permission from Elsevier Science.

Injection of capsaicin (2–80 nmol) into the preoptic area of the hypothalamus causes an abrupt hypothermic response [50], and increases the activity of warm-sensitive neurons while depressing the activity of cold-sensitive neurons [30]. In addition, capsaicin (~4 μM) can evoke glutamate release in rat hypothalamic slice preparations [13,22] and enhance postsynaptic currents [51] (Fig. 2).

### Thalamus

Nociceptive (pinch sensitive) neurons in the medial thalamus can be activated by arterial capsaicin infusion [52-54], an effect that can be blocked by morphine [54]. Interestingly, TRPV1 gene knockout mice are not impaired in the detection of noxious mechanical stimuli as determined by tail pinch, von-Frey test, and spinal nociceptive neuron responses [12]. However, this does not rule out the possibility that thalamic TRPV1 receptors modulate noxious mechanical information once it has been detected.

### Ventral tegmental area

**Figure 3:**
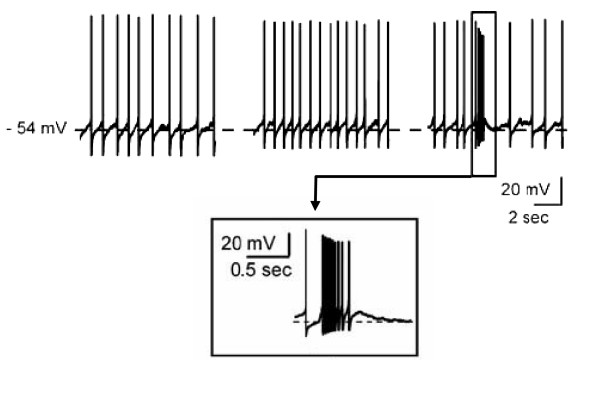
**TRPV1 modulates spike activity of ventral tegmental area**. Dopamine cell recorded in whole cell current clamp mode in-vitro slice preparation. Bath application of capsaicin (middle trace) augmented firing frequency of ventral tegmental cells. In some cases bursting activity was observed (right-most trace and inset). Inset also shows action potentials superimposed on depolarizing envelope. These results indicate that TRPV1 activation can directly modulate central neurons. Adapted from [55] ^© ^2005 with permission from Macmillan Publishers Ltd.

Application of capsaicin (1–10 μM) to the ventral tegmental area increases the firing rate and bursting activity of dopaminergic neurons in-vitro [55] (Fig. 3). In addition, it was shown that activation of TRPV1 receptors of the ventral tegmental area could enhance dopaminergic output to the nucleus accumbens, following peripheral noxious stimulation [55].

### Substantia nigra

Peripheral administration of capsaicin results in bursting activity in the substantia nigra, recorded with intracortical electroencephalogram in rats [45]. Injection of capsaicin into the substantia nigra can enhance locomotor behaviors (100 nmol capsaicin) and produce peripheral vasodilation (30–100 nmol capsaicin) [56,57]. In-vitro studies have demonstrated that TRPV1 activation (1–10 μM capsaicin) enhances glutamatergic synaptic transmission to dopaminergic neurons of the substantia nigra [58]. In addition, analysis of excitatory postsynaptic currents suggested a presynaptic mechanism for this enhancement [58]. Importantly, this study demonstrated that TRPV1 is activated by endogenous ligands in-vitro, since antagonism of the receptor reduced the frequency of spontaneous excitatory postsynaptic currents.

### Hippocampus

In the hippocampal CA1 region, TRPV1 activation enhances paired-pulse depression [7,59]. It is possible that the mechanism of the depression was the activation of presynaptic TRPV1 receptors at GABAergic terminals, which feed back and inhibit CA1 neurons [59,60]. This would be expected to have the net effect of increasing GABA output [60]. However, TRPV1 receptor activation was found to inhibit the influx of calcium and reduce GABA release in synaptosomal hippocampus preparations [60]. This discrepancy between in-vitro and ex-vivo data may be a consequence of the disruption of intracellular or extracellular molecules under ex-vivo conditions. These molecules may modulate TRPV1 receptor function. Alternatively, the discrepancy may be due to rapid desensitization of TRPV1 receptors under ex-vivo conditions [60].

### Cerebellum

Microiontophoretically applied capsaicin into the cerebellum depresses neuron spike activity [61]. This finding is interesting because it differs from the excitatory effect of capsaicin in other regions of the brain. In contrast with the hypothalamus and cerebral cortex (below), activation of the TRPV1 receptor does not evoke glutamate release in cerebellum tissue slices [13].

### Cortex

The somatosensory cortex and ACC are both involved in the processing of pain [24,25,33,62]. However, few studies have examined the effect of direct TRPV1 receptor activation in these regions. It has been shown that activation of the TRPV1 receptor can evoke glutamate release from cortical slices [13]. In addition, a study by Toldi et al. [63] showed that local application of capsaicin to the somatosensory cortex reduced mechanically and electrically evoked potentials of anesthetized rats [63].

**Figure 4:**
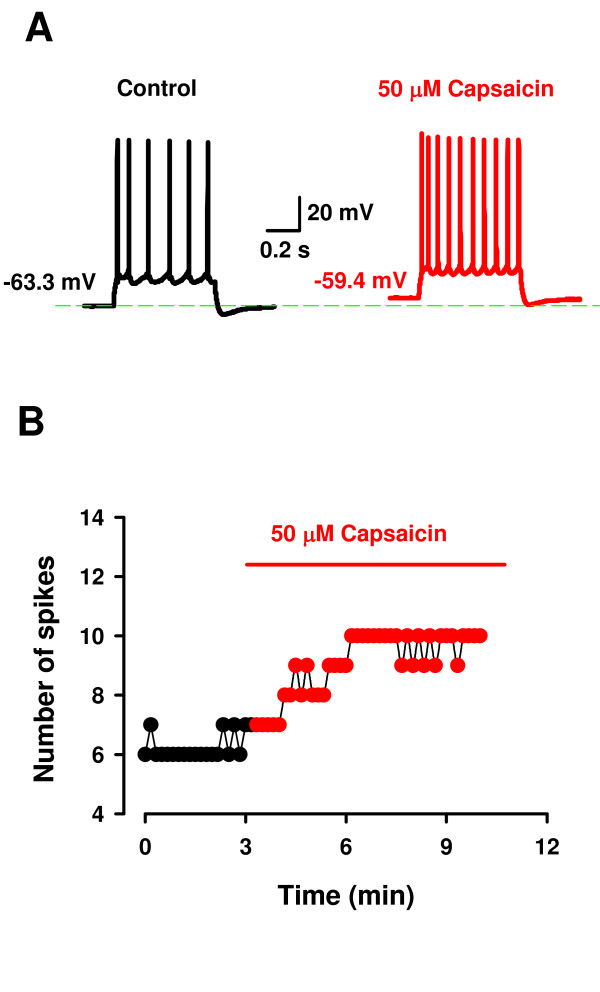
**Capsaicin activates anterior cingulate cortex neurons**. A. Current injection (220 pA, 800 ms) into a pyramidal neuron in layer II-III in the ACC induced action potential firing. Perfusion of capsaicin (50 μM) significantly increased the number of spikes. Note the slight depolarizing effect of capsaicin in the neuron (dashed line). The results indicate that TRPV1 receptors are functional in a cortical brain region involved in pain processing. B. Time course of capsaicin effect on the neuron shown in A

Preliminary in-vitro data from our laboratory show that capsaicin application (50 μM) to the ACC increases the firing frequency of some neurons (Fig. 4), while depressing firing of other neurons. The differing direction of neuron firing patterns is similar to the effects observed in the hypothalamic preoptic area [30]. Since the ACC is involved in the formation of pain-associated memory and the descending modulation of nociception [64,65], it will be of interest to determine whether or not the TRPV1 receptor can influence these processes.

## Future directions

The TRPV1 receptor is expressed and functional throughout the brain. It is possible that populations of TRPV1 receptors within the brain are also involved in processing nociceptive information. This not only suggests that local manipulation of cortical TRPV1 may lead to alterations in pain behaviors, but also warns against assigning a strictly peripheral role of TRPV1 receptors in pain transmission. For example, since TRPV1 receptor knockout mice have a global gene deletion, it is not possible to discount the role of the receptors expressed at supraspinal structures in the pain phenotypes reported [12].

The most prominent expression of TRPV1 mRNA and receptors is in the DRG with expression much less concentrated in supraspinal structures. While, minor expression does not necessarily imply minor functions, research will need to carefully examine the role of the TRPV1 receptors in the brain. Indeed, many early studies were conducted without determining the specificity of the capsaicin effect to the TRPV1 receptor. Future experimentation will need to confirm the involvement of TRPV1 receptors in the brain with antagonists and TRPV1-deficient mouse studies.

Electrophysiological studies indicate that the actions of the TRPV1 receptor in supraspinal structures are largely presynaptic [13,22,43,49,58]. However, this receptor is reported to be localized to postsynaptic spines in the brain [19]. This inconsistency is likely due to incomplete analysis of synaptic TRPV1 localization throughout the whole brain.

Direct activation of the TRPV1 receptor in different brain regions can result in diverse effects including changes in body temperature, respiration, heart rate, blood pressure and locomotion [27,28,41,42,46,50,56,57]. This indicates that TRPV1 receptor function depends on where it is located in the brain.

The TRPV1 receptor is not only of interest to the basic neuroscientist but also among pharmaceutical industries. The initial observation that there was little or no expression of this receptor in the brain suggested that this receptor would be an ideal target for the treatment of pain. Although treating pain through the modulation of the TRPV1 receptor is an exciting prospect, caution should be exercised when developing drugs to target this receptor since it is expressed and functional in the brain [3,14-21] and body [6,13]. More extensive research of supraspinal TRPV1 receptors is needed to determine its role in synaptic transmission and the control of behavior.

## Abbreviations

ACC Anterior cingulate cortex

ATP Adenosine triphosphate

DRG Dorsal root ganglion

NTS Solitary tract nucleus

TRPV1 Transient receptor potential vanilloid-1

CB1 Cannabinoid receptor-1

## Competing interests

The author(s) declare that they have no competing interests.

## Supplementary Material

Additional File 1 **TRPV1 detection in the brain**. TRPV1 can be detected in the brain using a variety of methodologies. Abbreviations as follows: d, detected but intensity not reported; [^3^H]RTX resiniferatoxin binding; IH, immunohistochemistry; ISH, in-situ hybridization; NB, northern blot, nd, not detected; RPA, ribonuclease protection assay; RT-PCR, reverse transcription polymerase chain reaction; WB, western blot; * to ***, relative intensity of detection; References are indicated at the top of each column. Click here for file
