# Supplementary material for: Hot receptors in the brain
Source: Mol Pain. 2006 Nov 8;2:34. doi: 10.1186/1744-8069-2-34 (PMC1647269; doi:10.1186/1744-8069-2-34)
Supplement: Additional File 1 — TRPV1 detection in the brain. TRPV1 can be detected in the brain using a variety of methodologies. Abbreviations as follows: d, detected but intensity not reported; [3H]RTX resiniferatoxin binding; IH, immunohistochemistry; ISH, in-situ hybridization; NB, northern blot, nd, not detected; RPA, ribonuclease protection assay; RT-PCR, reverse transcription polymerase chain reaction; WB, western blot; * to ***, relative intensity of detection; References are indicated at the top of each column. [file 1744-8069-2-34-S1.doc]

|  |  | **METHODOLOGY** | | | | | | |
| --- | --- | --- | --- | --- | --- | --- | --- | --- |
| **Region** | **Subregion** | **[^3^H]RTX**  **[18]/[17]/[20]/[15]** | **RPA**  **[21]** | **WB**  **[19]** | **NB**  **[3]** | **IH**  **[16]/[14]** | **ISH**  **[16]** | **RT-PCR**  **[16]/[22]/[1]** |
| *White matter* |  |  |  |  | nd |  |  |  |
|  | Corpus callosum | * |  |  |  |  |  |  |
| *Telencephalon* |  |  |  |  |  |  |  |  |
| Olfactory system |  |  |  | d | nd |  |  |  |
|  | Glomerular layer | *** |  |  |  |  |  |  |
|  | Olfactory nerve layer | *** |  |  |  |  |  |  |
|  | Piriform cortex 4 | * |  |  |  |  |  |  |
| Neocortex |  | *** | * | *** | nd |  |  | d / *** |
|  | Agranular insular cortex | *** |  |  |  | / d | *** |  |
|  | Frontal cortex | *** |  |  |  |  | *** |  |
|  | Granular insular | *** |  |  |  | / d | *** |  |
|  | Parietal cortex | *** |  |  |  |  | *** |  |
|  | Somatosensory cortex | / nd / * |  |  |  |  |  |  |
| Metacortex |  |  |  |  |  |  |  |  |
|  | Cingulate cortex | * |  |  |  | / d |  |  |
|  | Retrosplenial cortex | *** |  |  |  |  |  |  |
| Basal ganglia |  |  |  |  | nd |  |  |  |
|  | Caudate putamen | * |  |  |  |  |  |  |
|  | Globus pallidus | * |  |  |  |  |  |  |
|  | Core of the accumbens nucleus | ** |  | ** |  |  |  |  |
|  | Substantia nigra | * |  |  |  | d | *** |  |
|  | Substantia innominata | * |  |  |  |  |  |  |
|  | Striatum |  |  |  |  | * |  | / *** |
| Hippocampal formation |  |  | * |  | nd | d | *** | d / * |
|  | CA1 region | ** |  |  |  |  |  |  |
|  | CA2 region | ** |  |  |  |  |  |  |
|  | CA3 region | ** |  |  |  |  |  |  |
|  | Dentate gyrus | ** |  | *** |  | d | *** |  |
| Amygdala |  |  |  |  | nd |  |  |  |
|  | Amygdaloid nuclei | ** |  |  |  | d | * |  |
|  | Lateral septal nucleus | ** / / / nd |  |  |  | d |  |  |
|  | Medial septal nucleus | * |  |  |  |  |  |  |
| Epithalamus |  |  |  |  |  |  |  |  |
|  | Habenula | * |  |  |  | d | * |  |
| Thalamus |  |  |  |  | nd |  | * | / * |
|  | Paracentral thalamic nucleus | *** |  |  |  |  |  |  |
|  | Paraventricular thalamic nucleus | ** |  |  |  |  |  |  |
|  | Nucleus reuniens | ** |  |  |  |  |  |  |
|  | Reticular thalamic nucleus | * |  |  |  |  |  |  |
|  | Ventral posterior thalamic nucleus | * / * / * |  |  |  |  |  |  |
|  | Ventromedial thalamic nucleus | * / * / * |  |  |  |  |  |  |
|  | Zona incerta | * |  |  |  |  |  |  |
| Subthalamus |  |  |  |  | nd |  |  |  |
|  | Subthalamic nucleus | * |  |  |  |  |  |  |
| Hypothalamus |  | / * |  |  | nd | d | ******* | d / *** |
|  | Anterior hypothalamic area | * |  |  |  | * |  |  |
|  | Arcuate hypothalamic nucleus | ** |  |  |  | * |  |  |
|  | Dorsomedial hypothalamic nucleus | ** |  |  |  | * |  |  |
|  | Lateral hypothalamic area | * |  |  |  |  |  |  |
|  | Paraventricular hypothalamic nucleus region | * |  |  |  | * |  |  |
|  | Periventricular hypothalamic nucleus | ** |  |  |  |  |  |  |
|  | Ventromedial hypothalamic nucleus | ** |  |  |  |  |  |  |
|  | Preoptic Area | / * / ** |  |  |  |  |  |  |
|  | Suprachiasmatic nucleus |  |  |  |  |  | d |  |
| *Mesencephalon* |  |  |  | d | nd |  |  | / * |
|  | Interpeduncular nuclei | *** / / / nd |  |  |  | d |  |  |
|  | Periaqueductal grey | *** |  |  |  |  |  |  |
|  | Deep mesencephalic nucleus | * |  |  |  |  |  |  |
|  | Raphé nuclei | ** |  |  |  | d |  |  |
|  | Superior layer of superior colliculus | ** |  |  |  |  |  |  |
|  | Nucleus of spinal trigeminal tract | / / / *** |  |  |  | d | * |  |
| *Rhombencephalon* |  |  |  |  | nd |  |  | / * |
|  | Locus coeruleus | ** / ** / ** |  |  |  | d | *** |  |
|  | Olivary complex | ** |  |  |  | d | *** |  |
|  | Solitary tract nucleus | / / / *** |  |  |  | d | * |  |
| *Cerebellum* |  | / nd **/ *** | * | d |  |  | *** | / *** |
|  | Cerebellar cortex | *** |  |  |  |  |  |  |
|  | Cerebellar medulla | * |  |  |  |  |  |  |
|  | Granular cell layer | ** |  |  |  |  |  |  |
|  | Molecular cell layer | ** |  |  |  |  |  |  |
|  | Deep cerebellar nuclei | ** |  |  |  |  |  |  |
| *Spinal cord* |  |  | * | d | nd |  |  | d / nd |
|  | Dorsal horn | ** / *** / *** |  |  |  |  |  |  |
|  | Ventral horn | * |  |  |  |  |  |  |
